# Mast cell tryptase stimulates myoblast proliferation; a mechanism relying on protease-activated receptor-2 and cyclooxygenase-2

**DOI:** 10.1186/1471-2474-12-235

**Published:** 2011-10-14

**Authors:** Elise Duchesne, Marie-Hélène Tremblay, Claude H Côté

**Affiliations:** 1 CHUQ Research Center and Faculty of Medicine, Laval University, 2705 boul. Laurier, Québec, Québec, G1V 4G2, Canada

## Abstract

**Background:**

Mast cells contribute to tissue repair in fibrous tissues by stimulating proliferation of fibroblasts through the release of tryptase which activates protease-activated receptor-2 (PAR-2). The possibility that a tryptase/PAR-2 signaling pathway exists in skeletal muscle cell has never been investigated. The aim of this study was to evaluate whether tryptase can stimulate myoblast proliferation and determine the downstream cascade.

**Methods:**

Proliferation of L6 rat skeletal myoblasts stimulated with PAR-2 agonists (tryptase, trypsin and SLIGKV) was assessed. The specificity of the tryptase effect was evaluated with a specific inhibitor, APC-366. Western blot analyses were used to evaluate the expression and functionality of PAR-2 receptor and to assess the expression of COX-2. COX-2 activity was evaluated with a commercial activity assay kit and by measurement of PGF_2_α production. Proliferation assays were also performed in presence of different prostaglandins (PGs).

**Results:**

Tryptase increased L6 myoblast proliferation by 35% above control group and this effect was completely inhibited by APC-366. We confirmed the expression of PAR-2 receptor *in vivo *in skeletal muscle cells and in satellite cells and *in vitro *in L6 cells, where PAR-2 was found to be functional. Trypsin and SLIGKV increased L6 cells proliferation by 76% and 26% above control, respectively. COX-2 activity was increased following stimulation with PAR-2 agonist but its expression remained unchanged. Inhibition of COX-2 activity by NS-398 abolished the stimulation of cell proliferation induced by tryptase and trypsin. Finally, 15-deoxy-Δ-^12,14^-prostaglandin J_2 _(15Δ-PGJ_2_), a product of COX-2-derived prostaglandin D_2_, stimulated myoblast proliferation, but not PGE_2 _and PGF_2_α.

**Conclusions:**

Taken together, our data show that tryptase can stimulate myoblast proliferation and this effect is part of a signaling cascade dependent on PAR-2 activation and on the downstream activation of COX-2.

## Background

Injuries to the musculoskeletal system, such as muscle damage resulting from extensive and unaccustomed exercise, trauma, dystrophies, ischemia and toxin exposition, have important functional impacts and therefore very significant consequences for daily activities. The same can be said for the sarcopenia observed in the elderly and the cachexia typically associated with chronic systemic diseases. Although our general understanding of muscle damage and regeneration has progressed significantly, the exact role of various signals and cells implicated in the process is still unclear in skeletal muscle.

Following muscle damage, there is an orchestrated recruitment of inflammatory cells, which produce pro- and/or anti-inflammatory signals leading initially to the removal of the damaged tissue and subsequently to the resolution of inflammation and tissue repair [1-5]. The key steps between inflammation and muscle healing include the resolution of inflammation and activation of myoblasts, which are present as quiescent muscle precursor cells in the form of satellite cells and stem cells in adult myofibers. Activated myoblasts migrate to the site of injury, proliferate, differentiate and fuse into myotubes to form new myofibers, eventually leading to restoration of form and function of the damaged muscle. Macrophages, mast cells, endothelial cells, fibroblasts and muscle fibers themselves can produce growth and transcription factors that influence myoblasts during muscle regeneration. Indeed, prostaglandins (PGs) and several growth factors such as fibroblast growth factors (FGFs), insulin-like growth factor-1 (IGF-1), transforming growth factor beta (TGF-β), epidermal growth factor (EGF), stem cell factor (SCF), and hepatocyte growth factor (HGF) have been identified as potent mediators of myogenesis [6-12]. However, the regulatory processes controlling early myoblast activation and subsequent proliferation remain only partially understood in skeletal muscle.

Mast cells are derived from bone marrow and their role has long been considered to be restricted to host defense and allergic reactions. However, it is now well documented that they can also modulate inflammation onset and resolution and also the repair phase [1,13-15]. Increase in the density and the proportion of degranulated mast cells has been observed in skeletal muscle following various types of injury [1,16,17]. In models of bupivacaine-induced muscle injury and unloading-reloading muscle damage, mast cell stabilization caused a reduced neutrophil recruitment and a subsequent increase in the density of pro-inflammatory macrophages, confirming the implication of mast cells in modulating leukocyte infiltration [1,16]. While several studies have looked at the role of mast cells in the inflammatory period, their potential action on the myogenic process has been less investigated.

It is known that mast cells can contribute to tissue repair and sometimes fibrosis by activating proliferation of epithelial [18] and smooth muscle cells [19] as well as fibroblasts [20], through the release of tryptase, a serine protease [21]. The tryptase-initiated signaling cascade supporting this proliferative response has been elucidated in fibroblasts [22,23]. These studies have shown that tryptase modulates the proliferation of these cells through the cleavage of protease-activated receptor-2 (PAR-2) and a subsequent increase in COX-2 expression, a finding adding further importance to the role of COX-2 as an important actor for myogenesis in situation of skeletal muscle repair. There is now a long list of studies showing that COX-2 inhibition can impair skeletal muscle hypertrophy [24] and healing [25] processes and that COX-2 regulates growth of atrophied muscles [26] and early stages of skeletal muscle regeneration [27]. PAR-2 is a G-protein-coupled receptor whose activation requires the cleavage of the extracellular amino terminus of the receptor. PAR-2 receptor can respond to its known natural agonists such as tryptase and trypsin and also to synthetic agonists like SLIGKV. Whereas tryptase is specific to PAR-2 receptor, trypsin can also cleave and activate PAR-1 and -4 receptors. Data from one study suggest that synthetic PAR-2 agonists influence the proliferation of primary rodent neonatal myoblasts [28], but the effect of natural agonists and the mechanism underlying this phenomenon have not been documented and remain unknown.

Our objective was thus to document the existence of such mechanism in skeletal muscle cells. More specifically, we aimed to: 1) determine whether tryptase, via PAR-2 activation, stimulates L6 myoblast proliferation *in vitro *and 2) identify elements of the signaling cascade downstream of PAR-2. Our working hypothesis was that tryptase stimulates myoblast proliferation via cleavage of PAR-2 receptor and subsequent activation of COX-2. Our results confirmed the expression of PAR-2 receptor in L6 myoblasts and in satellite cells of mature rat skeletal muscle. In addition, we showed that activation of PAR-2 receptor leads to stimulation of L6 myoblast proliferation and that this effect is dependent on COX-2 activity.

## Methods

### Animals

Female Wistar rats weighing 125-150 g were used to determine the presence of PAR-2 in mature skeletal muscle. The treatment and care of these animals were approved by the Laval University Research Center Animal Care and Use Committee based on the guidelines of the Canadian Council on Animal Care.

### Muscle homogenization of rat extensor digitorum longus (EDL) for PAR-2 probing

EDL muscles were homogenized at 4°C in lysis buffer containing 20 mM Tris, pH 7.5, 140 mM NaCl, 1 mM MgCl_2_, 1 mM CaCl_2_, 10% glycerol, 1% Igepal, 2 mM Na_3_VO_4_, 8.3 mM NaF, 0.2 mM PMSF and a cocktail of protease inhibitors P3840 from Sigma-Aldrich (St-Louis (MO), USA) using using a Polytron. Protein content was determined using bicinchoninic acid (BCA) protein assay reagent (Thermo Fisher Scientific, Rockforf (IL), USA).

### Cell culture

Subconfluent cultures of immortalized L6 rat skeletal myoblasts (ATCC, Manassas (VA), USA) were maintained in α-Mimimum Essential Medium (α-MEM) (Gibco, Carlsbad (CA), USA) supplemented with 10% fetal bovine serum (FBS) (HyClone, Logan (UT), USA).

### Proliferation assay

For measurement of proliferation rate, cells were seeded onto 96-well plate at a density of 3,000 cells/well in 65 μL of α-MEM containing 1% FBS and treated with or without tryptase (Sigma-Aldrich; St-Louis (MO), USA), trypsin (Sigma-Aldrich), PAR-2 synthetic activating peptide SLIGKV (Bachem Americas; Torrance (CA), USA), or prostaglandins PGF_2_α, PGE_2 _or 15Δ-PGJ_2 _(Cayman Chemical; Ann Arbor (MI), USA), at various doses for 48 h. When testing tryptase inhibition, tryptase was pre-incubated with a specific tryptase inhibitor, APC-366 (kind gift from American Peptides; Vista, (CA), USA), for 30 min at 37°C before addition to cells while COX-2 inhibition was tested by pre-incubation with a specific COX-2 inhibitor, NS-398, under the same conditions. The media was renewed after the first 24 h of incubation. In all experiments, the number of viable cells at the end of the experiment was determined by the use of CellTiter 96 Aqueous One Solution cell proliferation assay (Promega, Madison (WI), USA) according to the protocol proposed by the manufacturer.

### Western Blot

For analysis of COX-2 protein expression, adherent L6 cells were stimulated by tryptase (4 mU/ml) for 60 min or lipopolysaccharide (LPS) (10 μg/ml) (Sigma-Aldrich, St-Louis (MO), USA) for 3 h. For phospho-ERK1/2 protein expression, cells were deprived of serum for 4 h before being stimulated by tryptase (4 mU/ml) pre-incubated or not for 30 min at 37 °C with APC-366 (300 μM) or with NS-398 (100 μM) for periods ranging from 10 to 120 min. Cells were lysed in the lysis buffer previously described and protein content was determined by BCA protein assay reagent. Immunoblots were performed as described [29] by using primary antibodies PAR-2 (dilution 1:500) (Santa Cruz Biotechnology, Santa Cruz (CA), USA), COX-2 (dilution 1: 1,000) (Santa Cruz Biotechnology, Santa Cruz (CA), USA), phospho-p44/42 MAPK (Erk1/2) (Thr202/Tyr204), p44/p42 MAPK (ERK1/2) (137F5) (dilution 1:1,000) (Cell Signaling Technology, Danvers (MA), USA) or GAPDH (1:1,000) (Santa Cruz Biotechnology, Santa Cruz (CA), USA) and their secondary antibodies which are anti-rabbit HRP conjugated (dilution 1:10,000) (Amersham, Buckinghamshire, England) for PAR-2, p44/42 MAPK and GAPDH, anti-goat HRP conjugated (dilution 1:10,000) (Santa Cruz Biotechnology) for COX-2 or anti-mouse HRP conjugated (dilution 1:10,000) (Santa Cruz Biotechnology) for phospho-p44/42 MAPK.

### Reverse transcription and quantitative PCR (Q-PCR) analysis

For analysis of COX-2 gene expression, L6 cells were seeded in α-MEM 1% for 3 h before being stimulated by tryptase (4 mU/ml) for 10 to 120 min. RNA extraction was performed using RNeasy mini kit (Qiagen, Hilden, Germany) following the manufacturer protocol. 4 μg of total RNA was used for cDNA synthesis using Superscript II reverse transcription kit (Invitrogen Corporation, Carlsbad (CA), USA) in recommended conditions, using 500 ng of random hexamers (Roche, Nonnenwald, Germany). Primers used to amplify cDNA were designed using the free software Primer 3 (by Steve Rozen and Helen J, Skaletsky) available online at http://frodo.wi.mit.edu/primer3/. For COX-2, they were S: 5'-CTGAGGGGTTACCACTTCCA-3' and AS: 5'-TGAGCAAGTCCGTGTTCAAG-3' and for the house-keeping gene GAPDH, they were S: 5'-AAACCCATCACCATCTTCCA-3' and AS: 5'-GTGGTTCACACCCATCACAA-3'. The sequences were further analyzed with the software Amplify 3X (http://engels.genetics.wisc.edu/amplify/index.html) and synthesized at our institution. Q-PCR was performed by using a 36-well Rotor-Gene-3000 thermal cycler (Corbett Research, Mortlake, New South Wales, Australia) and results were analyzed with Rotor Gene version 6.0.27 software. cDNA (4 μl) from each sample were amplified by gene-specific primers described above (100 nM of each primer) in a 20 μl reaction containing 500 mM dNTP (Roche, Nonnenwald, Germany), 3 mM MgCl_2 _(Sigma-Aldrich, St-Louis (MO), USA), SYBR^® ^Green (1/30,000 dilution) (Invitrogen Corporation, Carlsbad (CA), USA), 5% DMSO (Sigma-Aldrich, St-Louis (MO), USA) and 1 U Taq polymerase (Roche, Nonnenwald, Germany). Thermal profile was: 95°C (15 s), 63°C (20 s), 72°C (25 s); 35 cycles. Fluorescence produced by SYBR^® ^Green was measured at the end of the extension step of each cycle. At the end of the 35 cycles of amplification, the Melt^® ^Procedure was performed to determine the specificity of the amplification. The fold increase of gene expression of COX-2 above GAPDH was evaluated by 2^ΔΔCT ^relative quantification method described by Dussault et al. (2006) [30], where CT represents the cycle number where the fluorescence intensity reaches a threshold value in a PCR reaction expressed in the exponential phase.

### Immunohistochemistry

Transverse sections (10 μm thick) were obtained in the mid-belly section of the EDL muscle. Sections were adhered to positive charged microscope slides for immunostaining of PAR-2 and PAX-7 as previously described [31]. Briefly, sections were incubated with or without (negative control) primary anti-PAR-2 antibody (1:200) (Santa Cruz Biotechnology, Santa Cruz (CA), USA) or anti-PAX-7 antibody (1:100) overnight at 4°C and followed by a 1 h incubation with biotinylated anti-rabbit IgG and biotinylated anti-mouse IgG secondary antibodies (1:200), respectively. The monoclonal antibody PAX-7 developed by Kawakami, A. was obtained from the Developmental Studies Hybridoma Bank developed under the auspices of the NICHD and maintained by The University of Iowa, Department of Biology, Iowa City, IA 52242.

### Measurement of COX activity

COX-2 activity was assessed directly by the use of a commercial COX activity assay kit (Cayman Chemical), allowing measurement of total COX activity and of the relative contribution of the two isoforms (COX-1 and -2) when using the selective COX-1 inhibitor SC-560. Briefly, cells were seeded in α-MEM supplemented with 10% FBS until reaching 80-90% confluence and then incubated for 2 h with or without trypsin (200 nM) for 30 min at 37°C before performing the test according to the procedure proposed by the manufacturer. To further validate these measurements, COX activity was also assessed indirectly via measurement of PGF_2_α production. Cells were seeded in α-MEM supplemented with 1% FBS until reaching 80-90% confluence. Cells were then incubated for 12 h with trypsin (200 nM) or tryptase (2 mU/ml), or pre-incubated with indomethacin (10 μM) (Sigma) or NS-398 (100 μM) for 30 min at 37°C before the addition of PAR-2 agonist. Production of PGF_2_α was assayed by enzyme immunoassay using a custom-made competitive ELISA as described previously [32].

### Statistical analysis

Data are presented as means ± s.e.m. Data were analyzed using Tukey-Kramer HSD multiple-comparison test when significant F ratios were obtained following two-way ANOVAs. P < 0.05 was considered to be statistically significant.

## Results

### Expression and functionality of PAR-2 receptor

**Figure 1:**
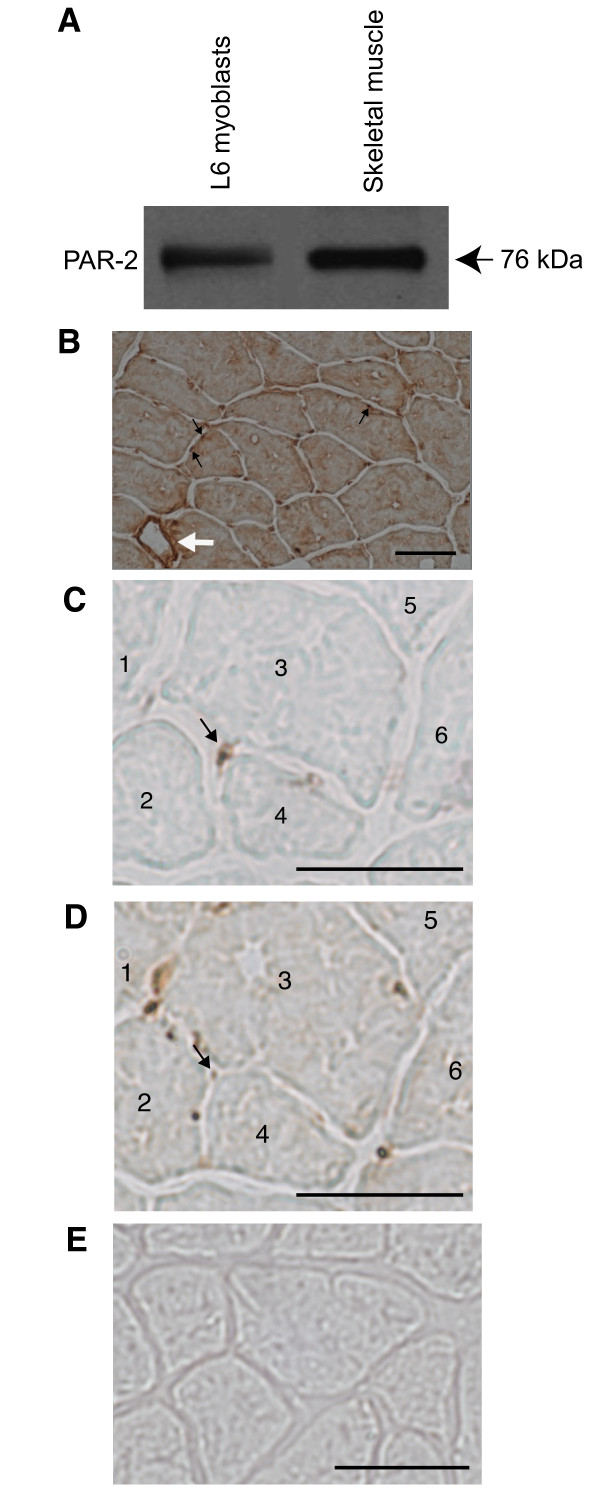
**PAR-2 receptor expression in L6 myoblasts, in myofibers and in satellite cells of skeletal muscle**. A) Expression of PAR-2 receptor in L6 myoblasts and skeletal muscle (EDL) by western blot (representative sample of 4 separate experiments). B) Detection of PAR-2 protein by immunohistochemistry on EDL muscle myofibers (black arrows) and blood vessel (white arrow). C) and D) Immunohistochemistry performed on two consecutive sections showed that satellite cells identified by PAX-7 staining (C) also expressed PAR-2 receptor (D). The numbered cells 1 to 6 served to the localization of satellite cell on consecutives sections. E) Negative control of an immunohistochemistry performed without primary antibody PAR-2 or PAX-7. Bar = 50 μm.

**Figure 2:**
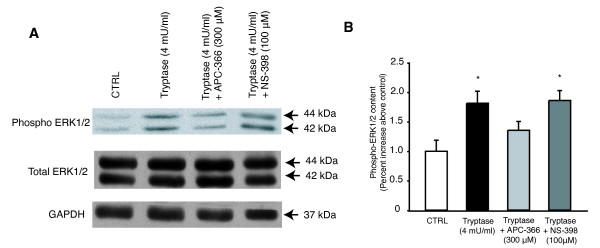
**PAR-2 receptor functionality in L6 myoblasts**. A) Levels of phospho-ERK1/2 and total ERK1/2 by western blot following stimulation with tryptase, tryptase pre-incubated with its inhibitor APC-366 or with COX-2 inhibitor NS-398. GAPDH served as loading control. B) Quantification of the level of phospho-ERK1/2. Each data point represents the mean of at least 4 separate experiments (mean ± s.e.m). * Significantly different from control (CTRL), *P *< 0.05.

The expression of PAR-2 receptor was detectable in L6 cells and in rat skeletal muscle (EDL) (Figure 1A). The assessment of PAR-2 distribution by immunohistochemistry showed that PAR-2 is expressed at the membrane level of muscle fibers (Figure 1B, black arrows) and in satellite cells, which were identified by PAX-7 staining (Figure 1C and 1D). Specificity of PAR-2 staining was validated by the intense staining observed around blood vessel since endothelial cells are known to express PAR-2 receptor [33] (Figure 1B, white arrow). In L6 cells stimulated with tryptase, the proportion of phosphorylated ERK1/2, a downstream indicator of PAR-2 activation [23,33], increased significantly compared to untreated cells whereas no significant response was seen when tryptase was pre-incubated with its specific inhibitor, APC-366 (Figure 2A and 2B). Pre-incubation with the COX-2 specific inhibitor NS-398 did not influence tryptase-induced phosphorylation of ERK1/2 (Figure 2A and 2B). These results confirmed that PAR-2: 1) is present and functional in L6 cells, 2) is expressed in satellite cells of mature skeletal muscle, and 3) can be activated by tryptase.

### Effect of tryptase, trypsin and activating peptide on L6 myoblast proliferation

**Figure 3:**
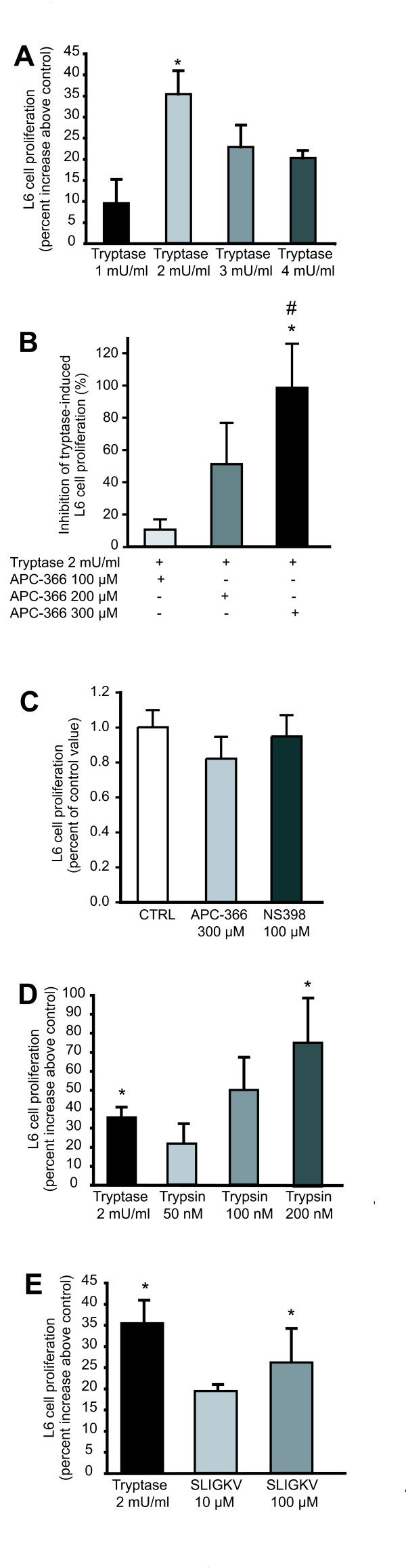
**Effect of PAR-2 agonists and tryptase inhibitor on L6 myoblast proliferation**. L6 myoblast proliferation following treatment with either A) tryptase, D) trypsin or E) PAR-2 synthetic activating peptide SLIGKV, while control wells received medium with 1% FBS only. B) Tryptase was pre-incubated with APC-366 where control wells (not pre-incubated with the inhibitor) represent 0% inhibition (not shown). C) L6 myoblasts were incubated either with APC-366 or NS-398 alone for 48 h to test for the possible cell toxicity. Each data point represents the mean of five separate experiments (mean ± s.e.m). * Significantly different from control (CTRL) and ^# ^significantly different from APC-366 100 μM, *P *< 0.05.

We next verified if PAR-2 activation by natural and synthetic agonists could stimulate cell proliferation. Tryptase increased proliferation of L6 cells by 35% above control at a concentration of 2 mU/ml (Figure 3A). Pre-incubation of tryptase with APC-366 caused a dose-dependent inhibition of the effect of tryptase on L6 cell proliferation (Figure 3B). L6 cell viability was not affected by APC-366 at the maximal concentration used (Figure 3C). To further confirm the implication of PAR-2 in the cascade modulating L6 myoblast proliferation, trypsin and the PAR-2 activating peptide SLIGKV were used. Both were found to stimulate L6 cell proliferation in a dose-dependent manner (Figure 3D, 3E). More precisely, trypsin increased proliferation by 76% while SLIGKV stimulated proliferation to a lower extent (26%). These results confirm that activation of PAR-2 receptor stimulates L6 myoblast proliferation.

### Implication of PAR-2 and COX-2 in L6 myoblast proliferation

**Figure 4:**
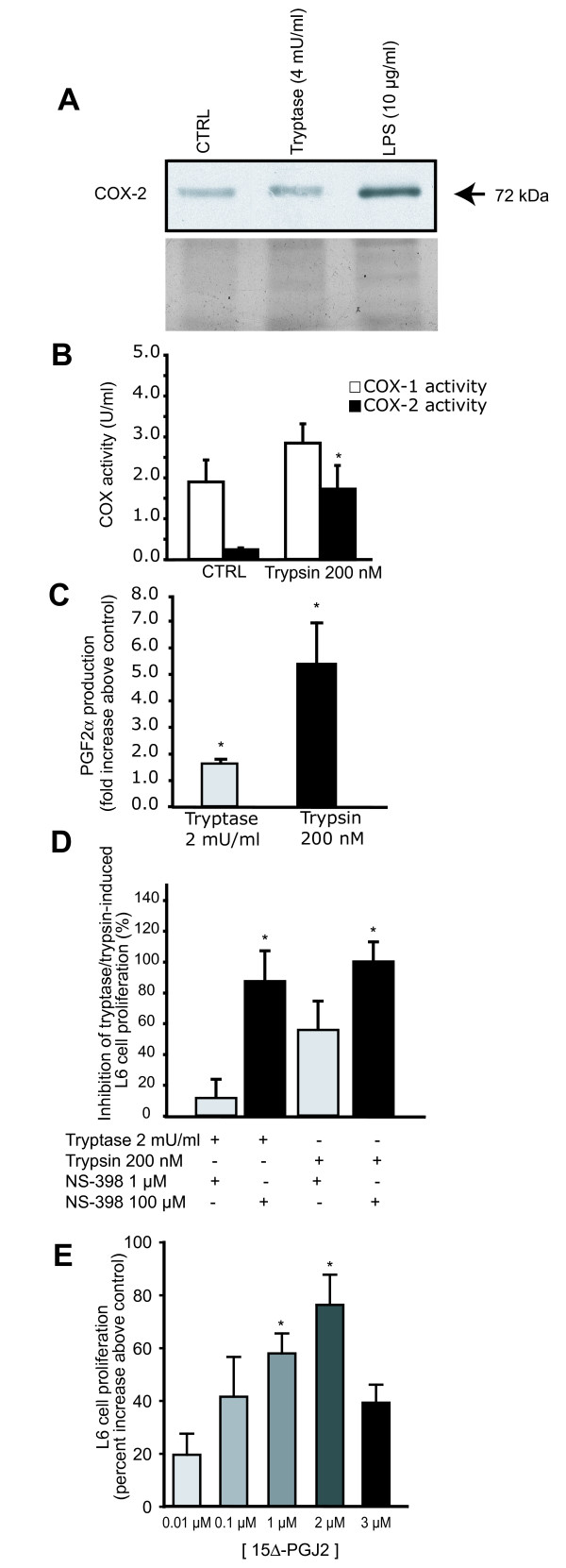
**COX-2 expression and activity in L6 myoblasts**. A) COX-2 protein content was determined by western blotting in L6 myoblasts under basal conditions (CTRL) or in cells treated with tryptase (representative sample of 4 separate experiments) or with LPS. B) COX-1 and COX-2 activity following trypsin stimulation. C) COX-2 activity was measured by the production of PGF_2_α through enzyme immunoassay following tryptase and trypsin stimulation. D) L6 myoblast proliferation following incubation with increasing doses of NS-398 in presence of tryptase or trypsin where control wells (not pre-incubated with the inhibitor) represent 0% inhibition (not shown). E) L6 myoblast proliferation following incubation with increasing doses of 15Δ-PGJ_2_, while control wells received medium only. Each data point represents the mean of five separate experiments (mean ± s.e.m). * Significantly different from control, *P *< 0.05.

Since it has been demonstrated in fibroblasts that COX-2 is part of the signaling cascade leading to the stimulation of proliferation following PAR-2 activation, we investigated its putative role in the modulation of L6 myoblast proliferation. COX-2 was found to be constitutively expressed in L6 myoblasts and contrary to what was seen in fibroblasts, we were unable to detect any modulation of its expression following tryptase stimulation (Figure 4A). The same conclusion was reached by using Q-PCR to look at COX-2 mRNA level (data not shown). However, these results did not exclude the possibility of a modulation of the activity of COX-2. Measurement of COX-2 activity revealed a 7.1 fold increase in L6 cells stimulated with trypsin, whereas COX-1 activity was not increased significantly (Figure 4B). To further validate this finding, we looked at PGF_2_α production in L6 cells following PAR-2 activation and found that tryptase and trypsin led respectively to a 1.6 and 5.4 fold stimulation above control (Figure 4C), whereas the COX inhibitors NS-398 (COX-2 specific) and indomethacin (COX-1 and -2) abolished this effect to the same extent (data not shown). As could be predicted based on these findings, inhibiting COX-2 activity with NS-398 very significantly blunted the effect of tryptase and trypsin on cell proliferation (Figure 4D), an effect that could not be explained by a toxic effect of NS-398 since it did not impact on cell viability at the concentrations used (Figure 3C).

The modulation of COX-2 activity following PAR-2 activation led us to test the influence of various PGs on L6 cell proliferation. Exogenous PGE_2 _and PGF_2_α did not have any effect on cell proliferation, but 15Δ-PGJ_2_, a final product of COX-2-derived PGD_2_, stimulated L6 cell proliferation in a dose-dependent manner (Figure 4E). Taken together, these results suggest that modulation of COX-2 activity, and not its expression, is an intricate part of the cascade modulating L6 myoblast proliferation following PAR-2 activation and that 15Δ-PGJ_2 _could be implicated.

## Discussion

Mast cells have been identified as an important actor in the inflammatory process following injury and allergic reactions. However, there is also strong evidence suggesting an implication of mast cells in tissue regeneration through stimulation of cell proliferation. For instance, mast cell tryptase and PAR-2 receptor signaling pathway can stimulate proliferation in some cell types and is even linked to uncontrolled cell proliferation in various diseases such as mastocytosis [34], fibrosis [22] and asthma [35]. While several observations point for a role of mast cells in processes governing inflammation and regeneration of skeletal muscle regeneration, the relevance of this signaling cascade in this tissue has yet to be addressed.

### PAR-2 expression and activation by various agonists

Although the presence of PAR-2 receptors in skeletal muscle cells was previously observed in primary rodent neonatal myoblasts [28], we show here that immortalized L6 cells and satellite cells of rat mature skeletal muscle also express PAR-2 receptor, which is the only known receptor for tryptase [36]. The functionality of the receptor was also confirmed by the increased phospho-ERK1/2 content following tryptase-induced PAR-2 activation. Phospho-ERK1/2 is considered as a surrogate marker of the activation of PAR-2 [33] and is associated with tryptase-induced PAR-2 activation in human fibroblasts [22]. Tryptase inhibitor strongly annihilated the increase in phospho-ERK1/2 and the proliferative effect supporting the specificity of PAR-2 activation by tryptase. Lastly, the failure of NS-398 to modulate the phosphorylation of ERK1/2 following tryptase-induced PAR-2 activation suggests that this step is downstream of PAR-2 activation and upstream of COX-2 activation. These observations are in line with data from Frungieri et al. (2005) who demonstrated in human fibroblasts that the initial events of the tryptase/PAR-2 signaling pathway leading to cell proliferation rely on the activation of ERK1/2 [23]. We conclude that a functional PAR-2 receptor is expressed in L6 cells and that it can be stimulated by its two natural agonists and this leads to activation of ERK1-2.

Trypsin, a natural activator of PAR-2 [28], has been shown to mimic the proliferative effect of tryptase [37-39]. In our study, trypsin was a more potent stimulator of cell proliferation than tryptase, a finding previously observed [40]. One possible explanation for this difference is that trypsin is a PAR-1, -2 and -4 agonist while tryptase is PAR-2 specific. PAR-1 is expressed in myoblasts before fusion and PAR-1 agonists are able to stimulate proliferation of primary myoblasts [41]. As a result, we believe the response observed with trypsin in these experiments may have been the summation of both PAR-1 and -2 stimulations. The PAR-2 synthetic activating peptide (SLIGKV), on the other hand, induced a smaller response than tryptase and trypsin on L6 cells. This lower potency of SLIGKV is likely related to the inefficient presentation of the soluble peptide to its receptor binding domain leading to a less efficient stimulation compared to other molecules and/or to a shorter half-life in cell culture medium [36]. Another hypothesis to explain the difference between these three agonists is that PAR signaling can show 'functional selectivity' or 'biased agonism', a phenomenon leading to different signaling responses following activation of the same receptor by different agonists. Such behavior has been demonstrated for PAR-2 [42]. This phenomenon suggests that the effect observed following synthetic agonist stimulation does not correspond to a physiological response and reinforces the novelty of our study which documents the effect of natural PAR-2 agonists on skeletal muscle cell proliferation.

### PAR-2 activation stimulates L6 myoblast proliferation through a COX-2 dependent mechanism

In fibroblasts, cells with a very low constitutive expression of COX-2, activation of PAR-2 stimulates cell proliferation and leads to a very significant increase in the expression of COX-2 protein [22]. In L6 cells, we found a substantial constitutive expression of COX-2 protein, which was not modulated by stimulation of PAR-2 with tryptase. Constitutive COX-2 expression has also been reported in primary myoblast cultures and COX-2 activity was detected at basal level in these cells [26]. Interestingly, we also found that COX-2 is constitutively expressed in L6 myotubes and C2C12 myoblasts and myotubes (data not shown). COX-2 thus appears to be a constitutive component of not only proliferating myoblasts but also of differentiating myotubes in these two cell lines, observations again supporting its importance in muscle cell physiology.

The present data demonstrate that specific inhibition of COX-2 activity by NS-398 totally abolished the effect of tryptase and trypsin on L6 cell proliferation, a finding consistent with the growing number of papers showing that COX-2 inhibition can impact on skeletal muscle hypertrophy [24] and repair [25,27] and primary satellite cell proliferation [43]. However, in most of these papers, the outcome measures following COX-2 inhibition were mainly at the functional level with no attempt at identifying the signaling cascade. In the present study, we show a functional effect of COX-2 inhibition on cell proliferation that we were able to link to a signaling cascade. The crucial role of COX-2 in this cascade was further confirmed by the direct assessment of COX activity which revealed that a concentration of trypsin stimulating cell proliferation led to a very significant increase in COX-2 activity. Because specific COX-2 inhibitor (NS-398) and non-specific COX-1 and -2 inhibitor (indomethacin) induced the same level of inhibition on PGF_2_α production and total COX activity, we conclude that only COX-2 is involved. Overall, these experiments solidly confirm the implication of COX-2 activity in the signaling cascade. This finding can be integrated to results showing that COX-2 derived PGs are important for myogenesis and essential for the regulation of muscle growth in stretch-induced myoblast proliferation [44] and in skeletal muscle hypertrophy [24] and regeneration following injury [27] and atrophy [26]. Pavlath et al. previously demonstrated that the COX-2 pathway regulates early stages of myofiber growth during muscle regeneration. They postulated that attenuated myonuclear addition caused by COX-2 inhibition could explain the impact on growth: since myonuclear addition depends on satellite cell activation and proliferation, we propose that the PAR-2 cascade we depict here is the link between COX-2 activity and myoblast activation and proliferation.

To further investigate the role of COX-2 activity, we tested the effect of three PGs on cell proliferation. Our observations showed that only 15Δ-PGJ_2 _stimulated L6 cell proliferation while PGF_2_α and PGE_2 _were found to be without effect. Evidence supporting the role of 15Δ-PGJ_2 _in the resolution phase of inflammation is growing [45-47]; furthermore, it was shown to stimulate human fetal foreskin fibroblast proliferation [22]. In our model, the stimulating effect induced by 15Δ-PGJ_2 _was very significant and further underlines the importance of COX-2 activity. 15Δ-PGJ_2 _is traditionally known to exert its effect through PPARγ [22] and is considered its natural ligand. PPARγ expression can be detected in whole extracts of skeletal muscle [48,49], but its specific expression in myoblasts has not been reported so far and the consensus is that it is not expressed in myocytes. However, there is growing evidence that 15Δ-PGJ_2 _can have PPARγ-independent effects. For example, 15Δ-PGJ_2 _can inhibit TNFα production by covalent binding of IκB kinase, thus keeping the transcription factor NF-κB in an inactive state [46,50]. 15Δ-PGJ_2 _can also act directly via DP1 receptor to accomplish its anti-inflammatory effects [47]. We must thus conclude that the effect of 15Δ-PGJ_2 _on myoblast proliferation is likely PPARγ-independent and further work will be required to identify the target.

## Conclusions

In summary, we showed that the PAR-2 agonist tryptase can modulate L6 myoblast proliferation which involves the modulation of COX-2 activity and possibly 15Δ-PGJ_2 _production. These data suggest that mast cells, via tryptase release, could be one of the components in the process triggering the proliferation of myoblasts, as it is the case with other cells such as fibroblasts, epithelial cells and smooth muscle cells. In addition, this study reveals that the largely reported influence of COX-2 on muscle regeneration may be explained, at least in part, by its implication in this signaling cascade supporting skeletal muscle cell proliferation. Clinically speaking, these data should be added to an extensive list of publications showing that cells classically limited to the inflammatory phase are also important for the regeneration phase of tissue repair.

## Competing interests

The authors declare that they have no competing interests.

## Authors' contributions

ED participated in the design of the study, carried out all the experiments in the study and drafted the manuscript. MHT participated in the proliferation assays and helped to draft some sections of the manuscript. CHC conceived the study, participated in its design and coordination and helped to draft of the manuscript of the study. All authors read and approved the final manuscript.

## Pre-publication history

The pre-publication history for this paper can be accessed here:

http://www.biomedcentral.com/1471-2474/12/235/prepub

## References

[B1] Dumont N Lepage K Cote CH Frenette J Mast cells can modulate leukocyte accumulation and skeletal muscle function following hindlimb unloading J Appl Physiol 2007 103 1 97 104 10.1152/japplphysiol.01132.2006 17395758

[B2] Peake J Nosaka K Suzuki K Characterization of inflammatory responses to eccentric exercise in humans Exerc Immunol Rev 2005 11 64 85 16385845

[B3] Smith C Kruger MJ Smith RM Myburgh KH The inflammatory response to skeletal muscle injury: illuminating complexities Sports Med 2008 38 11 947 969 10.2165/00007256-200838110-00005 18937524

[B4] Tidball JG Inflammatory cell response to acute muscle injury Med Sci Sports Exerc 1995 27 7 1022 1032 10.1249/00005768-199507000-00011 7564969

[B5] Toumi H F'Guyer S Best TM The role of neutrophils in injury and repair following muscle stretch J Anat 2006 208 4 459 470 10.1111/j.1469-7580.2006.00543.x 16637872 PMC2100203

[B6] Allen RE Boxhorn LK Regulation of skeletal muscle satellite cell proliferation and differentiation by transforming growth factor-beta, insulin-like growth factor I, and fibroblast growth factor J Cell Physiol 1989 138 2 311 315 10.1002/jcp.1041380213 2918032

[B7] Allen RE Sheehan SM Taylor RG Kendall TL Rice GM Hepatocyte growth factor activates quiescent skeletal muscle satellite cells in vitro J Cell Physiol 1995 165 2 307 312 10.1002/jcp.1041650211 7593208

[B8] Doumit ME Cook DR Merkel RA Fibroblast growth factor, epidermal growth factor, insulin-like growth factors, and platelet-derived growth factor-BB stimulate proliferation of clonally derived porcine myogenic satellite cells J Cell Physiol 1993 157 2 326 332 10.1002/jcp.1041570216 8227164

[B9] Sheehan SM Allen RE Skeletal muscle satellite cell proliferation in response to members of the fibroblast growth factor family and hepatocyte growth factor J Cell Physiol 1999 181 3 499 506 10.1002/(SICI)1097-4652(199912)181:3<499::AID-JCP14>3.0.CO;2-1 10528236

[B10] Tatsumi M Kishi Y Miyata T Numano F Transforming growth factor-beta(1) restores antiplatelet function of endothelial cells exposed to anoxia-reoxygenation injury Thromb Res 2000 98 5 451 459 10.1016/S0049-3848(00)00190-0 10828485

[B11] Yablonka-Reuveni Z Balestreri TM Bowen-Pope DF Regulation of proliferation and differentiation of myoblasts derived from adult mouse skeletal muscle by specific isoforms of PDGF J Cell Biol 1990 111 4 1623 1629 10.1083/jcb.111.4.1623 2211828 PMC2116257

[B12] Zalin RJ The role of hormones and prostanoids in the in vitro proliferation and differentiation of human myoblasts Exp Cell Res 1987 172 2 265 281 10.1016/0014-4827(87)90386-7 3308494

[B13] Metz M Grimbaldeston MA Nakae S Piliponsky AM Tsai M Galli SJ Mast cells in the promotion and limitation of chronic inflammation Immunol Rev 2007 217 304 328 10.1111/j.1600-065X.2007.00520.x 17498068

[B14] Galli SJ Tsai M Mast cells: versatile regulators of inflammation, tissue remodeling, host defense and homeostasis J Dermatol Sci 2008 49 1 7 19 10.1016/j.jdermsci.2007.09.009 18024086 PMC2788430

[B15] Kalesnikoff J Galli SJ New developments in mast cell biology Nat Immunol 2008 9 11 1215 1223 10.1038/ni.f.216 18936782 PMC2856637

[B16] Cote CH Tremblay MH Duchesne E Lapoite BM Inflammation-induced leukocyte accumulation in injured skeletal muscle: role of mast cells Muscle Nerve 2008 37 6 754 763 10.1002/mus.20998 18506708

[B17] Gorospe JR Nishikawa BK Hoffman EP Recruitment of mast cells to muscle after mild damage J Neurol Sci 1996 135 1 10 17 10.1016/0022-510X(95)00255-Z 8926490

[B18] Cairns JA Walls AF Mast cell tryptase is a mitogen for epithelial cells. Stimulation of IL-8 production and intercellular adhesion molecule-1 expression J Immunol 1996 156 1 275 283 8598474

[B19] Chambers LS Black JL Poronnik P Johnson PR Functional effects of protease-activated receptor-2 stimulation on human airway smooth muscle Am J Physiol Lung Cell Mol Physiol 2001 281 6 L1369 1378 11704532 10.1152/ajplung.2001.281.6.L1369

[B20] Abe M Kurosawa M Ishikawa O Miyachi Y Effect of mast cell-derived mediators and mast cell-related neutral proteases on human dermal fibroblast proliferation and type I collagen production J Allergy Clin Immunol 2000 106 1 Pt 2 S78 84 10887338 10.1067/mai.2000.106058

[B21] Payne V Kam PC Mast cell tryptase: a review of its physiology and clinical significance Anaesthesia 2004 59 7 695 703 10.1111/j.1365-2044.2004.03757.x 15200544

[B22] Frungieri MB Weidinger S Meineke V Kohn FM Mayerhofer A Proliferative action of mast-cell tryptase is mediated by PAR2, COX2, prostaglandins, and PPARgamma: Possible relevance to human fibrotic disorders Proc Natl Acad Sci USA 2002 99 23 15072 15077 10.1073/pnas.232422999 12397176 PMC137545

[B23] Frungieri MB Albrecht M Raemsch R Mayerhofer A The action of the mast cell product tryptase on cyclooxygenase-2 (COX2) and subsequent fibroblast proliferation involves activation of the extracellular signal-regulated kinase isoforms 1 and 2 (erk1/2) Cell Signal 2005 17 4 525 533 10.1016/j.cellsig.2004.09.017 15601629

[B24] Novak ML Billich W Smith SM Sukhija KB McLoughlin TJ Hornberger TA Koh TJ COX-2 inhibitor reduces skeletal muscle hypertrophy in mice Am J Physiol Regul Integr Comp Physiol 2009 296 4 R1132 1139 10.1152/ajpregu.90874.2008 19176887 PMC4043321

[B25] Shen W Prisk V Li Y Foster W Huard J Inhibited skeletal muscle healing in cyclooxygenase-2 gene-deficient mice: the role of PGE2 and PGF2alpha J Appl Physiol 2006 101 4 1215 1221 10.1152/japplphysiol.01331.2005 16778000

[B26] Bondesen BA Mills ST Pavlath GK The COX-2 pathway regulates growth of atrophied muscle via multiple mechanisms Am J Physiol Cell Physiol 2006 290 6 C1651 1659 10.1152/ajpcell.00518.2005 16467402

[B27] Bondesen BA Mills ST Kegley KM Pavlath GK The COX-2 pathway is essential during early stages of skeletal muscle regeneration Am J Physiol Cell Physiol 2004 287 2 C475 483 10.1152/ajpcell.00088.2004 15084473

[B28] Chinni C de Niese MR Jenkins AL Pike RN Bottomley SP Mackie EJ Protease-activated receptor-2 mediates proliferative responses in skeletal myoblasts J Cell Sci 2000 113 Pt 24 4427 4433 11082036 10.1242/jcs.113.24.4427

[B29] Schagger H von Jagow G Tricine-sodium dodecyl sulfate-polyacrylamide gel electrophoresis for the separation of proteins in the range from 1 to 100 kDa Anal Biochem 1987 166 2 368 379 10.1016/0003-2697(87)90587-2 2449095

[B30] Dussault AA Pouliot M Rapid and simple comparison of messenger RNA levels using real-time PCR Biol Proced Online 2006 8 1 10 10.1251/bpo114 16446781 PMC1352391

[B31] Dumont N Bouchard P Frenette J Neutrophil-induced skeletal muscle damage: a calculated and controlled response following hindlimb unloading and reloading Am J Physiol Regul Integr Comp Physiol 2008 295 6 R1831 1838 10.1152/ajpregu.90318.2008 18784335

[B32] Asselin E Goff AK Bergeron H Fortier MA Influence of sex steroids on the production of prostaglandins F2 alpha and E2 and response to oxytocin in cultured epithelial and stromal cells of the bovine endometrium Biol Reprod 1996 54 2 371 379 10.1095/biolreprod54.2.371 8788188

[B33] al-Ani B Saifeddine M Hollenberg MD Detection of functional receptors for the proteinase-activated-receptor-2-activating polypeptide, SLIGRL-NH2, in rat vascular and gastric smooth muscle Can J Physiol Pharmacol 1995 73 8 1203 1207 10.1139/y95-172 8564891

[B34] Bunimovich O Grassi M Baer MR Systemic mastocytosis: classification, pathogenesis, diagnosis, and treatment Cutis 2009 83 1 29 36 19271568

[B35] Page S Ammit AJ Black JL Armour CL Human mast cell and airway smooth muscle cell interactions: implications for asthma Am J Physiol Lung Cell Mol Physiol 2001 281 6 L1313 1323 11704524 10.1152/ajplung.2001.281.6.L1313

[B36] Ossovskaya VS Bunnett NW Protease-activated receptors: contribution to physiology and disease Physiol Rev 2004 84 2 579 621 10.1152/physrev.00028.2003 15044683

[B37] Matsushima R Takahashi A Nakaya Y Maezawa H Miki M Nakamura Y Ohgushi F Yasuoka S Human airway trypsin-like protease stimulates human bronchial fibroblast proliferation in a protease-activated receptor-2-dependent pathway Am J Physiol Lung Cell Mol Physiol 2006 290 2 L385 395 16199437 10.1152/ajplung.00098.2005

[B38] Meyer-Hoffert U Rogalski C Seifert S Schmeling G Wingertszahn J Proksch E Wiedow O Trypsin induces epidermal proliferation and inflammation in murine skin Exp Dermatol 2004 13 4 234 241 10.1111/j.0906-6705.2004.00159.x 15086339

[B39] Ohta T Shimizu K Yi S Takamura H Amaya K Kitagawa H Kayahara M Ninomiya I Fushida S Fujimura T Protease-activated receptor-2 expression and the role of trypsin in cell proliferation in human pancreatic cancers Int J Oncol 2003 23 1 61 66 12792776

[B40] Bunnett NW Protease-activated receptors: how proteases signal to cells to cause inflammation and pain Semin Thromb Hemost 2006 32 Suppl 1 39 48 16673265 10.1055/s-2006-939553

[B41] Suidan HS Niclou SP Dreessen J Beltraminelli N Monard D The thrombin receptor is present in myoblasts and its expression is repressed upon fusion J Biol Chem 1996 271 46 29162 29169 10.1074/jbc.271.46.29162 8910573

[B42] Soh UJ Dores MR Chen B Trejo J Signal transduction by protease-activated receptors Br J Pharmacol 160 2 191 203 10.1111/j.1476-5381.2010.00705.x PMC2874842 20423334

[B43] Mendias CL Tatsumi R Allen RE Role of cyclooxygenase-1 and -2 in satellite cell proliferation, differentiation, and fusion Muscle Nerve 2004 30 4 497 500 10.1002/mus.20102 15372441

[B44] Otis JS Burkholder TJ Pavlath GK Stretch-induced myoblast proliferation is dependent on the COX2 pathway Exp Cell Res 2005 310 2 417 425 10.1016/j.yexcr.2005.08.009 16168411

[B45] Hong HY Jeon WK Kim BC Up-regulation of heme oxygenase-1 expression through the Rac1/NADPH oxidase/ROS/p38 signaling cascade mediates the anti-inflammatory effect of 15-deoxy-delta 12,14-prostaglandin J2 in murine macrophages FEBS Lett 2008 582 6 861 868 10.1016/j.febslet.2008.02.012 18291107

[B46] Prasad R Giri S Singh AK Singh I 15-deoxy-delta12,14-prostaglandin J2 attenuates endothelial-monocyte interaction: implication for inflammatory diseases J Inflamm (Lond) 2008 5 14 10.1186/1476-9255-5-14 PMC2531100 18691416

[B47] Rajakariar R Hilliard M Lawrence T Trivedi S Colville-Nash P Bellingan G Fitzgerald D Yaqoob MM Gilroy DW Hematopoietic prostaglandin D2 synthase controls the onset and resolution of acute inflammation through PGD2 and 15-deoxyDelta12 14 PGJ2 Proc Natl Acad Sci USA 2007 104 52 20979 20984 10.1073/pnas.0707394104 18077391 PMC2409252

[B48] Loviscach M Rehman N Carter L Mudaliar S Mohadeen P Ciaraldi TP Veerkamp JH Henry RR Distribution of peroxisome proliferator-activated receptors (PPARs) in human skeletal muscle and adipose tissue: relation to insulin action Diabetologia 2000 43 3 304 311 10.1007/s001250050048 10768091

[B49] Braissant O Foufelle F Scotto C Dauca M Wahli W Differential expression of peroxisome proliferator-activated receptors (PPARs): tissue distribution of PPAR-alpha, -beta, and -gamma in the adult rat Endocrinology 1996 137 1 354 366 10.1210/en.137.1.354 8536636

[B50] Powell WS 15-Deoxy-delta12,14-PGJ2: endogenous PPARgamma ligand or minor eicosanoid degradation product? J Clin Invest 2003 112 6 828 830 12975467 10.1172/JCI19796 PMC193674

